# Genome-Wide Identification and Characterization of Banana Ca^2+^-*ATPase* Genes and Expression Analysis under Different Concentrations of Ca^2+^ Treatments

**DOI:** 10.3390/ijms231911914

**Published:** 2022-10-07

**Authors:** Ronghui Ma, Na Tian, Jiashui Wang, Minlei Fan, Bin Wang, Pengyan Qu, Shiyao Xu, Yanbing Xu, Chunzhen Cheng, Peitao Lü

**Affiliations:** 1 College of Horticulture, Fujian Agriculture and Forestry University, Fuzhou 350002, China; 2 College of Horticulture, Shanxi Agricultural University, Taigu 030801, China; 3 Shaanxi Rural Science and Technology Development Center, Xi’an 710000, China; 4 Key Laboratory of Genetic Improvement of Bananas, Hainan Province, Haikou Experimental Station, Chinese Academy of Tropical Agricultural Sciences, Haikou 570102, China

**Keywords:** banana, Ca^2+^-ATPase, gene expression, Ca^2+^ deficiency, gene family

## Abstract

Ca^2+^-*ATPases* have been confirmed to play very important roles in plant growth and development and in stress responses. However, studies on banana (*Musa acuminata*) Ca^2+^-*ATPases* are very limited. In this study, we identified 18 Ca^2+^-*ATPase* genes from banana, including 6 P-IIA or ER (Endoplasmic Reticulum) type Ca^2+^-*ATPases* (*MaEACs*) and 12 P-IIB or Auto-Inhibited Ca^2+^-*ATPases* (*MaACAs*). The MaEACs and MaACAs could be further classified into two and three subfamilies, respectively. This classification is well supported by their gene structures, which are encoded by protein motif distributions. The banana Ca^2+^-ATPases were all predicted to be plasma membrane-located. The promoter regions of banana Ca^2+^-*ATPases* contain many *cis*-acting elements and transcription factor binding sites (TFBS). A gene expression analysis showed that banana Ca^2+^-*ATPases* were differentially expressed in different organs. By investigating their expression patterns in banana roots under different concentrations of Ca^2+^ treatments, we found that most banana Ca^2+^-*ATPase* members were highly expressed under 4 mM and 2 mM Ca^2+^ treatments, but their expression decreased under 1 mM and 0 mM Ca^2+^ treatments, suggesting that their downregulation might be closely related to reduced Ca accumulation and retarded growth under low Ca^2+^ and Ca^2+^ deficiency conditions. Our study will contribute to the understanding of the roles of Ca^2+^-*ATPases* in banana growth and Ca management.

## 1. Introduction

Calcium (Ca^2+^), one of the most important essential elements and secondary messengers in plants, plays a very important role in regulating the various stages of plants, such as growth and development and responses to various stresses [1]. For the maintenance of the Ca^2+^ level balance in cytosolic and the rapid, transient responses to both biotic and abiotic stresses, plants have evolved a series of mechanisms [2]. Ca^2+^-ATPases have been confirmed to play very important roles in sensing the transient Ca^2+^ level changes and in the maintenance of cytosolic Ca^2+^ homeostasis [3]. According to their subcellular localizations and structures, Ca^2+^-ATPases can be further divided into P-IIA or ER (Endoplasmic Reticulum) type Ca^2+^-ATPases (ECAs) and P-IIB or Auto-Inhibited Ca^2+^-ATPases (ACAs) [4]. Ca^2+^-ATPases are highly conserved in plants [5]. The ACA subfamily member usually contains an N-terminal auto-inhibitory domain which is required for the high affinity calmodulin (CaM) binding, whereas the ECAs lack the N-terminal domain [6,7]. Moreover, the ER-located ECAs are of high specificity to the substrate ATP, while the membrane localized ACAs can hydrolyze both GTP and ITP [8].

The *Lycopersicon LeECA1* gene was the first Ca^2+^-*ATPase* gene cloned from plant. Its expression level increased dramatically under 50 mM NaCl condition [9]. From then on, Ca^2+^-*ATPases* were continuously discovered in many plants and were found to be widely involved in many cellular and physiological processes of plants. For instance, the *Arabidopsis* (*Arabidopsis thaliana*) *AtACA9* was reported to be required for normal pollen tube growth and fertilization. Pollens of the *ataca9* mutant showed much lower growth potential, higher aborted fertilization frequency and a >80% reduction of seed set [10]. *AtACA10* has been reported to play an important role in regulating reproductive development, including adult phase growth and inflorescence development [11]. Moreover, the *ateca1*-*1* mutant displayed Ca^2+^ deficiency symptoms such as smaller plant size, shorter roots, small yellowish leaves and lack of bolts [12] under low Ca^2+^ conditions (0.2 to 0.4 mM). Similarly, the *ateca3* mutant was slightly more sensitive than the wild-type when Ca^2+^ was omitted from the medium, and the fresh weight of *ateca3-1* was significantly lower than that of the wild type plants [13].

In addition to their roles in plant growth and development, Ca^2+^-*ATPases* have also been repeatedly found to participate in plant stress responses. The *AtACA8* gene is low temperature inducible, and the *ataca8* mutant showed increased cold resistance and the higher expression of *CBFs*, *CAMTA* and *ZAT12* transcription factor genes, as well as cold responsive genes such as *UGE2* and *GolS3* [14]. In rice (*Oryza sativa* L.), *OsACA6* can improve plant salinity and drought stress tolerance by modulating the ROS scavenging pathway and enhancing the expression of stress-responsive genes [15]. *Glycine Soja GsACA1*-overexpression alfalfa plants exhibited higher tolerance to both carbonate alkaline and neutral salt stresses, and lower levels of membrane permeability and malondialdehyde (MDA) content, higher levels of superoxide dismutase (SOD) activity, proline (Pro) concentration and chlorophyll content under stress conditions [16]. Under Ca^2+^ deficiency and toxicity conditions, the expression of *Triticum aestivum TaECA1* and *TaECA3* significantly increased in both roots and shoots. And the expression levels of *TaACA2*, *3* and *4* in plants grown under Ca^2+^ deficiency and toxicity conditions differed significantly from plants grown under control conditions [3].

Banana (*Musa* spp.) is one of the most important fruit crops in the world. As a gigantic herb, the nutrient requirement of a banana tree is huge. However, due to the high temperature and rainy climate characteristic of their cultivation areas, banana trees often suffer greatly from various nutrient deficiencies [17]. Previous studies have shown that Nitrogen (N), Phosphorus (P), Potassium (K), Calcium (Ca), Magnesium (Mg), Sulfur (S) or Boron (B) deficiency in banana would result in leaf etiolation and root growth retardation [18]. Ca^2+^ is crucial for the growth and development of banana plants, and Ca^2+^ deficiency will cause malformation or carving of new banana leaves, significantly reduce the accumulation of dry matter in pseudostems and leaves, and inhibit growth of the whole banana plant [19]. The proper application of Ca fertilizer can improve photosynthesis ability and promote the growth and development of banana plants [20]. The crucial roles of Ca^2+^-*ATPases* in plant development and stress responses have been demonstrated in many species, but studies on banana Ca^2+^-*ATPases* genes is very limited. To provide a basis for future studies and applications of banana Ca^2+^-*ATPases*, genome-wide identification of this gene family was performed, their sequence characteristics were studied using a series of bioinformatics analyses, and their expression patterns in different parts of banana (including root, corm, pseudostem and leaf) were investigated using transcriptome data. Furthermore, we evaluated the effects of different concentrations of Ca^2+^ treatments on the growth of banana seedlings by measuring the aboveground part and root fresh weight, root number and root length, and the P, K, Ca, Mg and S contents in root, leaf and pseudostem. Moreover, to reveal the possible roles of Ca^2+^-*ATPases* in response to different Ca^2+^ levels, their expression patterns in banana roots under different concentrations of Ca^2+^ treatments (4 mM, 2 mM, 1 mM and 0 mM) were studied using a quantitative real time PCR (qRT-PCR). The results obtained in this study will be helpful for the understanding of the characteristics of the banana Ca^2+^-*ATPase* gene family and can provide insights into their roles in banana growth and development and stress responses.

## 2. Results

### 2.1. Identification and Characterization of Banana Ca^2+^-ATPase Gene Family Members

In total, 18 Ca^2+^-*ATPase* genes were identified from *M. acuminata*, including six *MaEACs* and 12 *MaACAs* (Table 1). According to their chromosomal location information, they were named as *MaECA1*~*MaECA6* and *MaACA1*~*MaACA12*, respectively. Among them, *MaACA9* and *MaACA12* have two variable transcripts, *MaACA3* has five transcripts, and these variable transcripts were named as *MaACA9*-*1* and *MaACA9*-*2*, *MaACA12*-*1* and *MaACA12*-*2*, and *MaACA3*-*1*~*3*-*5*, respectively. Banana Ca^2+^-ATPases consist of 942 to 1103 amino acids (aa) with a molecular weight ranging from 102,943.61 Da to 120,673.88 Da. The theoretical isoelectric point (pI) of Ca^2+^-ATPase proteins ranges from 5.19 to 8.87, and all of the MaECAs and seven of the MaACAs are acidic proteins, while the other five MaACAs (MaACA1, 2, 4, 11 and 12) are basic proteins. Their instability index ranges from 30.90 to 40.33. All of the banana Ca^2+^-ATPases are hydrophobic proteins with 6~11 transmembrane structures but without signal peptide. Protein subcellular localization prediction results showed that all banana Ca^2+^-ATPases were plasma membrane-located. 

**Figure 1:**
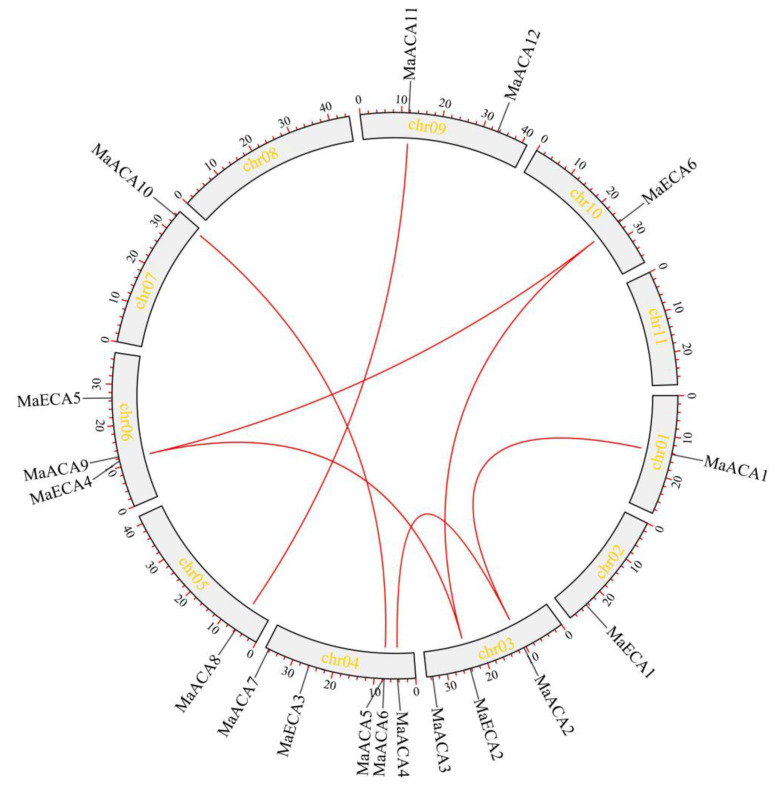
Chromosome localization and collinear relationship of banana Ca^2+^-*ATPase* genes. The red line indicates the segmental duplication gene pairs.

Banana Ca^2+^-*ATPase* genes are unevenly distributed on nine chromosomes (Figure 1). The most abundant of banana Ca^2+^-*ATPase* members are observed in chr04, with five members, followed by chr03, 06 and 09, with three, three, and two members, respectively, while chr01, 02, 07 and 10 each have only one member. Among the banana Ca^2+^-*ATPases*, there are seven segmental duplication gene pairs (*MaACA1*/*MaACA2*, *MaACA2*/*MaACA4*, *MaACA6*/*MaACA10*, *MaACA8*/*MaACA11*, *MaECA2*/*MaECA4*, *MaECA2*/*MaECA6* and *MaECA4*/*MaECA6*) and no tandem duplication pair (Figure 1). 

### 2.2. Phylogenetic Analysis of Ca^2+^-ATPase Genes

**Figure 2:**
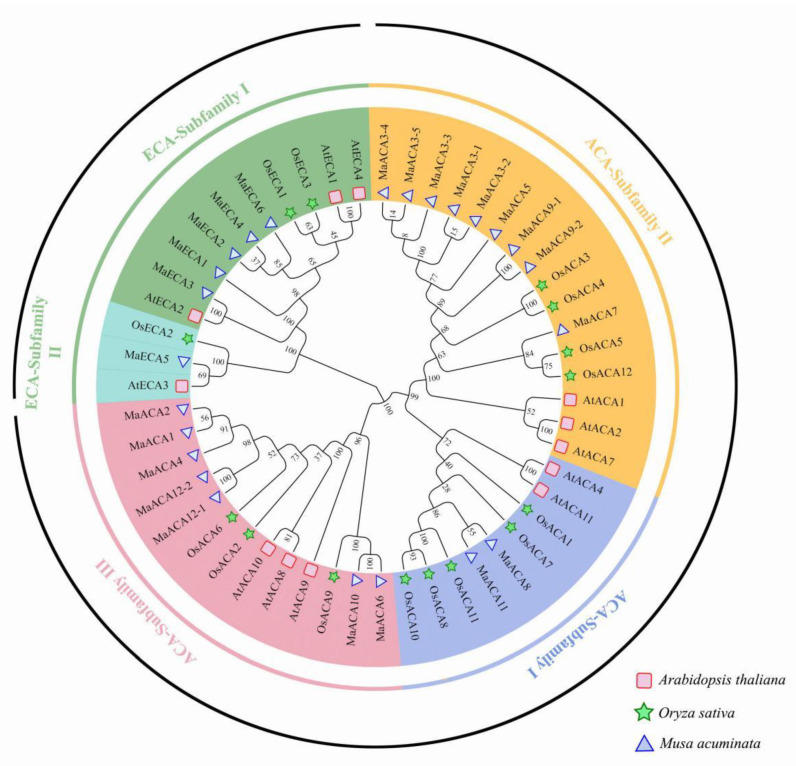
Phylogenetic analysis results based on the Ca^2+^-ATPases from *Arabidopsis thaliana* (At), *Oryza sativa* (Os) and *Musa acuminata* (Ma). Subfamily I-Subfamily V represents different Ca^2+^-ATPase subfamily, respectively.

By using the Ca^2+^-ATPase protein sequences from Banana (18), *Arabidopsis* (15) and Rice (15), the phylogenetic relationships among Ca^2+^-ATPases were analyzed (Figure 2). Results showed that the Ca^2+^-ATPases could be divided into two groups, the ACA group and the ECA group, which could be further classified into three and two subfamilies, respectively. ACA Subfamily I-III included two, four and six banana Ca^2+^-ATPase members, and ECA Subfamily I-II included five and one banana Ca^2+^-ATPase members, respectively.

### 2.3. Conserved Motif and Gene Structure Analysis Results

**Figure 3:**
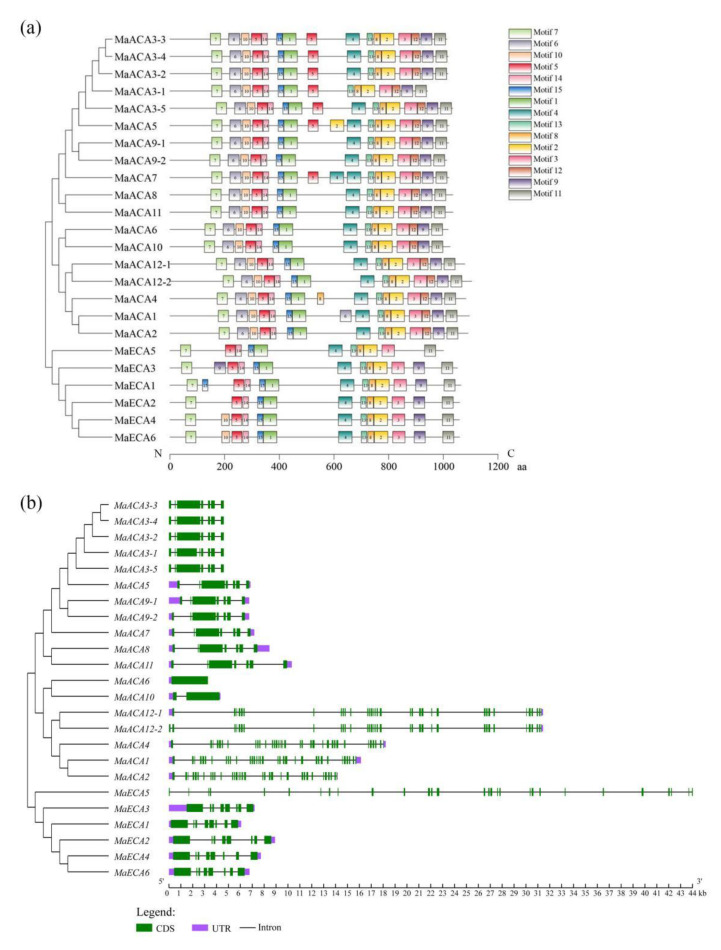
Motif distributions (**a**) in banana Ca^2+^-ATPase proteins and gene structures (**b**) of their corresponding genes.

A total of 15 motifs were identified in banana Ca^2+^-ATPases by using MEME (Figure 3a). A conserved motif analysis showed that all MaACAs were highly conserved (Figure 3a). Fourteen conserved motifs, except for motif 4, were found in all MaACAs. In addition, MaACA1 Subfamily III members contain two motif 6, and MaACA4 (Subfamily III) contain two motif 8. All of the MaACA1 Subfamily II members except for MaACA9-1 and MaACA9-2, contain two motif 5. Furthermore, MaACA5 (belonging to Subfamily II) contains two motif 2, while MaACA7 (Subfamily II) contains two motif 4. Motif 6 and 12 are lacking in both MaECA4 and MaECA6 of ECA Subfamily I. Motif 6, 10 and 12 are lacking in three ECA Subfamily I members, MaECA1, MaECA2 and MaECA3. In addition, MaECA1 (belonging to Subfamily I) contains two motif 15, and MaECA3 (belonging to Subfamily I) contains two motif 9. All the ECA members except MaECA5 contain all the identified motifs.

The exon-intron structures of banana Ca^2+^-*ATPase* genes from the same subfamily are found to be very similar in length and distribution (Figure 3b), indicating that they might originate from the same ancestor gene. The ACA subfamily I member genes have seven exons. All the ACA subfamily II members except *MaACA3*-*1* (8 exons) have seven exons. The number of exons in ACA subfamily III genes varies greatly. *MaACA6* has one exon, *MaACA10* has two exons, *MaACA12*-*2* has 35 exons and the other members have 34 exons. All members in ECA group I have eight exons, and the number of exons of *MaECA5* in ECA Group II was 29. 

### 2.4. Transcription Factor Binding Site (TFBS) and Cis-Acting Elements in Banana Ca^2+^-ATPase Promoters

**Figure 4:**
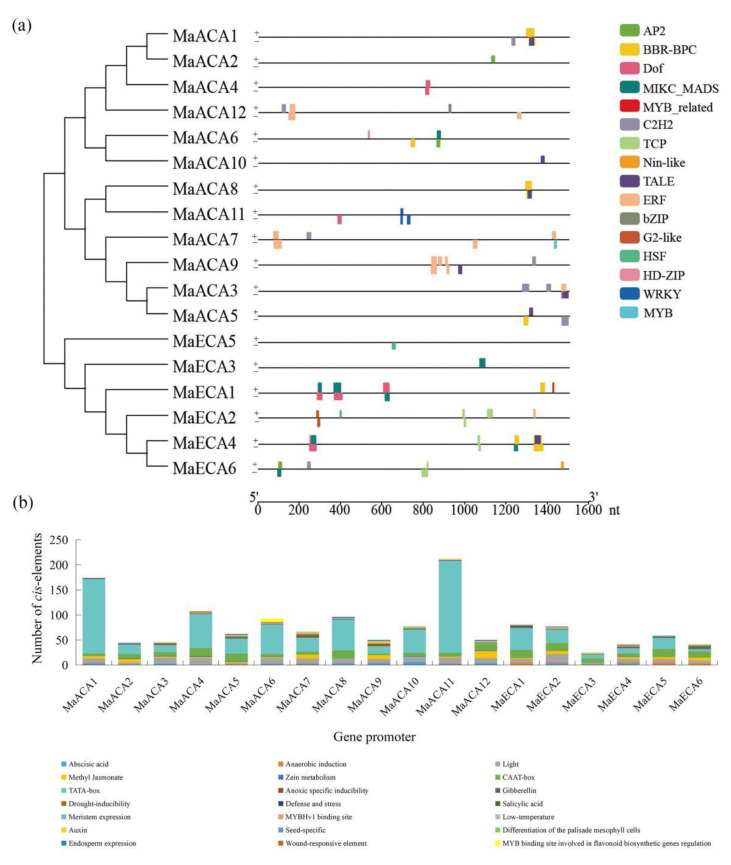
The predicted transcription factor binding sites (**a**) and *cis*-acting elements (**b**) on the promoters of banana Ca^2+^-*ATPase* genes.

In total, binding sites for 16 TF families (AP2, BBR-BPC, bZIP, C2H2, Dof, ERF, G2-like, HD-ZIP, HSF, MIKC-MADS, TALE, MYB, TCP, WRKY, MYB-related and Nin-like) were identified in the promoters of 7, 6, 1, 7, 4, 5, 1, 1, 2, 7, 7, 13, 1, 1, 1 and 3 family members, respectively. The binding sites for ERFs were found to be the largest in banana Ca^2+^-*ATPase* promoters, high up to 80, while the binding site number for bZIP, HD-ZIP, MYB, MYB-related and Nin-like was only one. In addition, there are significant differences in the TFBS number and distribution in the promoter regions of different banana Ca^2+^-*ATPases*. For instance, 59 TFBSs were found in *MaECA1*, while there is only one TFBS in the promoter region of *MaACA2* and *MaACA10* (Figure 4a). 

*Cis*-acting element prediction results showed that 21 kinds of *cis*-acting elements were identified in the banana Ca^2+^-*ATPase* promoters, which can be further classified into five categories, including light-responsive, core elements, phytohormone-responsive, stress-responsive and growth and development related elements. Light responsive elements and core box (TATA-box and CAAT-box) are present in all promoters of banana Ca^2+^-*ATPases*. Among the phytohormone-responsive elements, the number of elements related to abscisic acid (ABA) and methyl jasmonate (MeJA) was the largest, both containing 15 elements, followed by gibberellin (GA), auxin and salicylic acid (SA) related elements, which contain 12, 11 and 8 elements, respectively. In addition, there are several types of stress-responsive elements on the banana Ca^2+^-*ATPase* promoters, including anaerobic induction, low-temperature, drought-inducibility and defense and stress, which account for 10, 10, 8 and 3 elements, respectively. Furthermore, many plant growth and development related *cis*-elements were also found in the banana Ca^2+^-*ATPase* promoters, including meristem expression, zein metabolism, endosperm expression, anoxic specific inducibility, wound responsive element, seed-specific, differentiation of the palisade mesophyll cells, MYB binding site involved in flavonoid biosynthetic genes regulation, and MYBHv1 binding site elements, accounting for 8, 8, 4, 5, 3, 1, 1, 1 and 5 elements, respectively (Figure 4b).

### 2.5. Expression Profiles of Ca^2+^-ATPase Genes in Different Banana Parts

**Figure 5:**
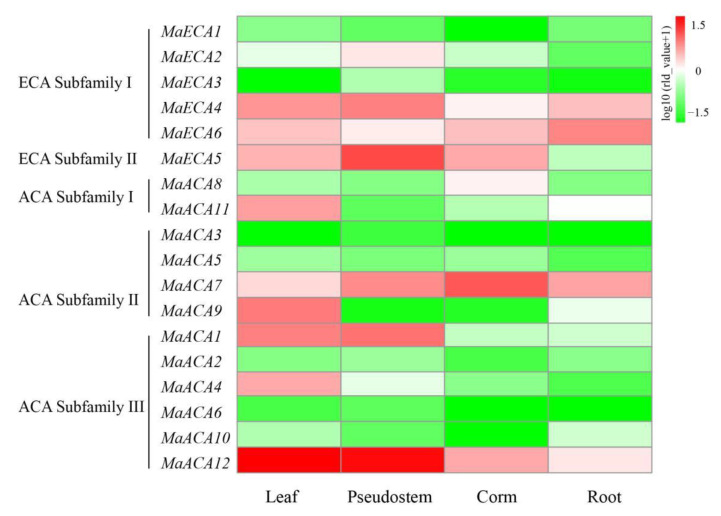
Heatmap for the expression of Ca^2+^-*ATPase* gene family members in different tissues. Red and green colors represent high and low expression levels, respectively.

According to our transcriptome data, the Ca^2+^-*ATPase* genes showed divergent expression patterns in different banana parts (Figure 5). In leaf, *MaACA12* (belonging to ACA Subfamily III) was predominantly expressed, followed by *MaACA1* (belonging to ACA Subfamily III), *MaACA9* (belonging to ACA Subfamily II). In pseudostem, *MaACA12* (belonging to ACA Subfamily III) was predominantly expressed, followed by *MaECA5* (belonging to ECA Subfamily II), *MaACA1* (belonging to ACA Subfamily III). *MaACA7* (belonging to ACA Subfamily II), *MaACA12* (belonging to ACA Subfamily III) and *MaECA5* (belonging to ECA Subfamily II) were highly expressed in corn. *MaECA6* (belonging to ECA Subfamily I), *MaACA7* (belonging to ACA Subfamily II) and *MaECA4* (belonging to ECA Subfamily I) were highly expressed in root. These results suggested that different Ca^2+^-*ATPase* family members might function differently in different organs.

### 2.6. Influences of Different Concentrations of Ca^2+^ Treatments on the Growth of Banana Seedling

**Figure 6:**
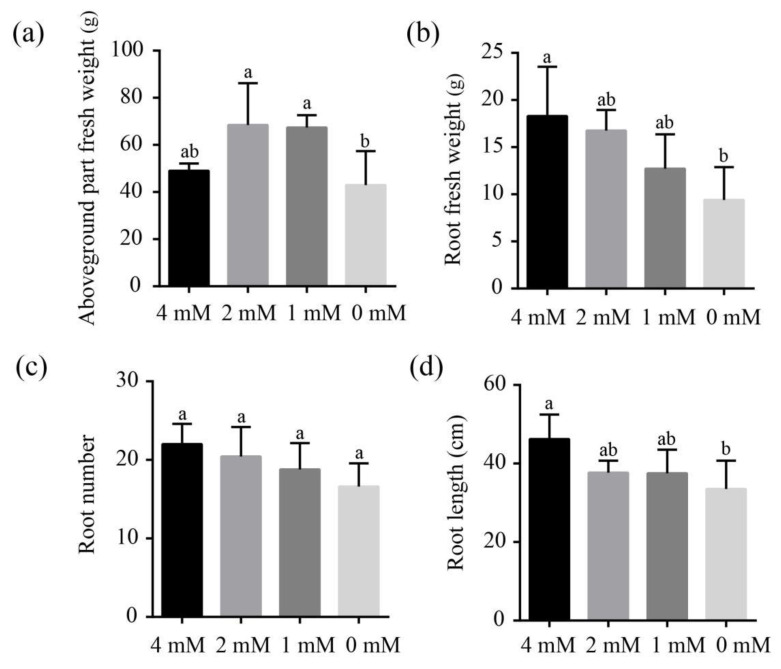
Effects of different concentrations of Ca^2+^ treatments on the aboveground part fresh weight (**a**), root fresh weight (**b**), root number (**c**) and root length (**d**). All data are displayed as mean ± standard deviation (SD) of at least three replicates. Different lowercase letters above columns represent significant difference at *p* < 0.05.

Six months post treatment with Hoagland solutions containing different concentrations of Ca^2+^ (4 mM, 2 mM, 1 mM and 0 mM), the growth-related parameters of river sand cultured banana seedlings were measured (Figure 6). The aboveground part fresh weight of banana plants from the 2 mM and 1 mM group was about 1.40-fold and 1.37-fold of that of the 4 mM group, respectively. The aboveground part fresh weight of 0 mM group decreased by about 12.40% compared with 4 mM concentration (Figure 6a). The root fresh weights of 2 mM, 1 mM and 0 mM groups were all lower than that of the 4 mM group, which decreased by 8.47%, 30.49% and 48.63%, respectively (Figure 6b). Similarly, the root number and root length of 2 mM, 1 mM and 0 mM groups were all lower than that of the 4 mM group, which decreased by 21.23%, 14.54% and 24.54%, and 18.46%, 18.79% and 27.49%, respectively (Figure 6c,d). These results indicate that insufficient Ca^2+^ supply greatly affected the growth and root development of banana plants.

### 2.7. Influences of Different Concentrations of Ca^2+^ Treatments on the P, K, Ca, Mg and S Contents in Banana Root, Leaf and Pseudostem

**Figure 7:**
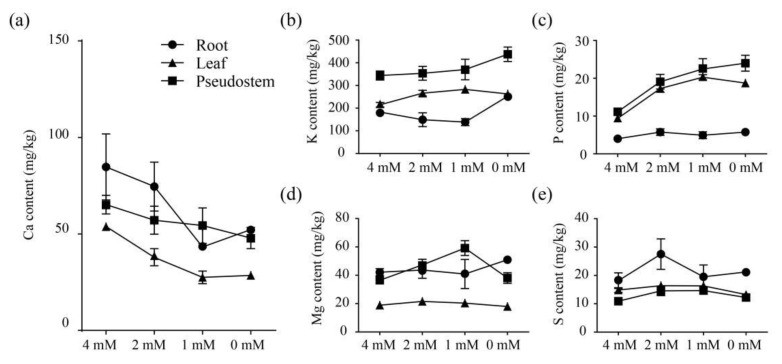
Effect of the different concentrations of Ca^2+^ on Ca (**a**), K (**b**), P (**c**), Mg (**d**) and S (**e**) content in root, leaf and pseudostem of banana seedlings. All data are displayed as mean ± standard deviation (SD) of at least three replicates.

The Ca content of the root, leaf and pseudostem decreased significantly with the reduction of Ca^2+^. The Ca content in root, leaf and pseudostem of the 2 mM group was only 88.02%, 70.40% and 87.68% of the 4 mM group, respectively. The Ca content in the three parts of the 1 mM group was only 51.07%, 51.09% and 83.33% of the 4 mM group, respectively. And the Ca content in the three tissues of the 0 mM group was only 61.64%, 52.83% and 73.47% of the 4 mM group, respectively (Figure 7a). Except the 1 mM group, the Ca content in root was lower than that in pseudostem, and the Ca content in different treatment groups all followed the order: root > pseudostem > leaf. Different concentrations of Ca^2+^ treatments also exhibited some influences on the K content in banana. Under 0 mM Ca^2+^ concentration treatment, the K content in banana leaf and pseudostem significantly increased (Figure 7b). The K content in different treatment groups all followed the order: pseudostem > leaf > root. With the decrease of Ca^2+^ concentrations, the P content in pesudostem increased and with the highest content in the 0 mM group, the P content in leaf showed a ‘rise-fall’ pattern and the highest P content was found in the 1 mM group. The P content in leaf and pseudostem of the 2 mM group is 1.84 and 1.71 times the 4 mM group, respectively. The P content in leaf and pseudostem of the 1 mM group is 2.16 and 2.02 times of the 4 mM group. And the P content in banana leaf and pseudostem of 0 mM group is 2.00 and 2.15 times the 4 mM group (Figure 7c). Moreover, it was found that the P content in root did not show obvious changes among different groups. The Mg content in pseudostems showed a ‘rise-fall’ pattern as the Ca^2+^ concentration decreased, and the highest Mg content was found in the 1 mM group. The Mg content in roots of the 0 mM group was the highest. The Mg content in leaves from different groups were found to be significantly lower than that in pseudostems and roots, and did not show significant difference among different groups (Figure 7d). The S contents in different banana parts all followed the order ‘root > leaf > pseudostem’ in all the treatment groups and the highest S contents were all found in the 2 mM group (Figure 7e).

### 2.8. The Expression Patterns of Banana Ca^2+^-ATPase Genes under Different Concentrations of Ca^2+^ Treatments

**Figure 8:**
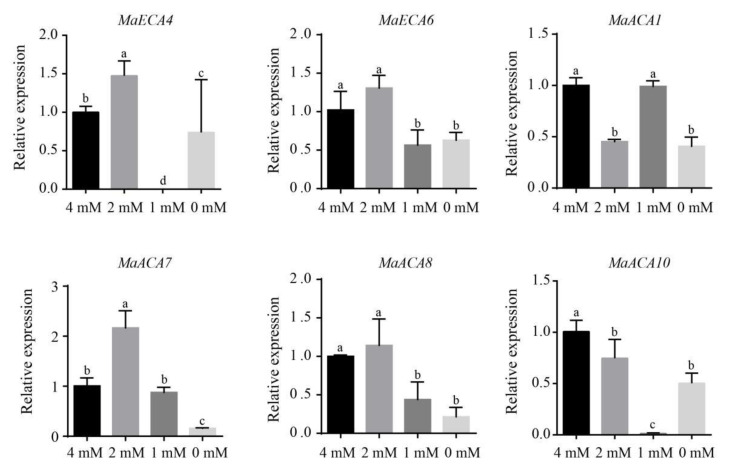
Quantitative real time PCR results of Ca^2+^-*ATPase* genes in banana roots under different concentrations of Ca^2+^ treatments. All data are displayed as mean ± standard deviation (SD) of three replicates. Different lowercase letters above the columns represent significant difference at *p* < 0.05.

We further studied the influences of different concentrations of Ca^2+^ treatments on the expression of six banana Ca^2+^-*ATPase* genes, including two *MaECAs* (*MaECA4* and *MaECA6*) and four *MaACAs* (*MaACA1*, *MaACA7*, *MaACA8* and *MaACA9*), using qRT-PCR (Figure 8). Results showed that the relative expression levels of all these genes except *MaACA1* in the 0 mM and 1 mM groups were lower than in the 2 mM and 4 mM groups. This indicated that both low Ca^2+^ (1 mM Ca^2+^) and Ca deficiency (0 mM Ca^2+^) suppressed the expression of banana Ca^2+^-*ATPases*. The expression levels of *MaECA4* and *MaACA7* in root of banana plants from the 2 mM group were significantly higher than that in the 4 mM group, but the expression of *MaACA1* and *MaACA10* were significantly lower than that in the 4 mM group. The expression of *MaECA4*, *MaECA6*, *MaACA8* and *MaACA10* in root of the 1 mM group were significantly lower than that in the 4 mM group. And the expression of *MaECA4* was the lowest among all the four groups.

## 3. Discussion

### 3.1. Comprehensive Genome-Wide Identification and Characterization of Banana Ca^2+^-ATPase Gene Family

In this study, we identified a total of 18 Ca^2+^-*ATPase* genes from *M. acuminata* genome, including six *MaEACs* and 12 *MaACAs*. The number of banana Ca^2+^-*ATPases* was higher than that of *Arabidopsis* (15) and rice (15) [21], but less than that in soybean (29) [16]. Gene duplication is considered to be a key factor contributing to the gene family expansion and gene function diversification [22]. In *Arabidopsis*, no gene duplication event was observed in the Ca^2+^-*ATPase* family [21]. One segmental duplication gene pair (*OsACA10* and *OsACA11*) and one tandem duplication gene pair (*OsACA2* and *OsACA3*) were identified in the rice genome [23]. In soybean, however, gene duplications occur in almost all the family members [16]. In our present study, seven segmental duplication gene pairs were identified, indicating that the expansion of the banana Ca^2+^-*ATPase* gene family was mainly caused by segmental duplications. 

Previous studies have revealed that plant Ca^2+^-ATPases are mainly localized in the plasma membrane as well as endo-membranes. *OsACA6*-GFP was exclusively observed in the plasma membrane [15]. In addition, AtECA3 has been reported to be localized in Golgi [13]. In our present study, all banana Ca^2+^-ATPases were predicted to be located in the plasma membrane, which was consistent with the results reported in some other plant species [4,10]. Protein subcellular location significantly determines the function of proteins. Therefore, it was predicted that the plasma membrane location of banana Ca^2+^-ATPases might be closely related to their functions in regulating intracellular Ca^2+^ homeostasis, which was achieved through transporting Ca^2+^ into extracellular space or sequestering Ca^2+^ in the ER and other organelles [24]. Similar to the gene structures of Ca^2+^-*ATPase* genes and conserved motifs of their encoded proteins in *Solanaceae* [25], *Medicago sativa* [26] and *Brassica rapa* [27], the banana Ca^2+^-ATPase members from the same subfamilies were found to be highly conserved. Moreover, the ACA group is found to be of higher conservation than the ECA group.

Transcription factors bind to the specific *cis*-acting elements and regulate the expression of a vast number of genes, which is an important epigenetic pathway [28]. In our study, many light responsive, core elements, phytohormone-responsive, stress responsive and growth and development related elements, as well as TFBSs for 16 kinds of transcription factors were found in the banana Ca^2+^-*ATPase* promoters. AP2/ERF, bZIP, C2H2, MYB and WRKY have been reported to play vital roles in regulating the growth, development and stress responses of banana [29,30,31]. Previous studies have shown that the promoter of *AtACA8* contains various TFBS responses to low temperature, in consistence, the gene’s expression is responsive to low temperature [14]. Thus, it was hypothesized that the Ca^2+^-*ATPase* gene expression was regulated by many TFs and responds to a variety of stresses.

### 3.2. Ca^2+^ Deficiency Impairs Banana Growth and Nutrient Homeostasis

After treating the banana plants with different concentrations of Ca^2+^ (4, 2, 1 and 0 mM) for six months, we investigated the influences of Ca^2+^ levels on the growth of banana. The banana seedlings grown under 2, 1 and 0 mM Ca^2+^ showed shorter and less roots compared to plants from the 4 mM group. Consistently, similar results were also reported in wheat [3]. It is widely known that Ca^2+^ is an essential plant nutrient [32]. Previous studies have shown that Ca^2+^ deficiency can cause leaf-rot/wilting phenotype in Maize (*Zea mays* L.) [33]. In cabbage (*Brassica oleracea* L. var. Capitata), the fresh matter of aerial parts decreased up to 37% in plants under Ca^2+^ deficiency [34]. Ca^2+^ deficiency also caused the reduction of plant biomass and root growth in trifoliate (*Poncirus trifoliate* L.) rootstock seedlings [35]. In our study, compared with the aboveground part, the roots showed more significant difference among different groups, which could be manifested by the significant reduction of root length and root fresh weight. In addition, our study showed that 2, 1 and 0 mM Ca^2+^ treatments greatly affected the macro-elements accumulations in banana root, pseudostem and leaf. An excess or deficiency of nutrients in soil will destroy the ion homeostasis in plants, resulting in plant nutritional imbalance and retarded plant growth. Previous studies reported that different concentrations of exogenous Ca^2+^ treatments could affect the contents of K, Mg, Fe, Mn, Cu and Zn in leaf and the pericarp of pomegranate (*Punica granatum* L.) [36]. In our study, the Ca content in banana root, pseudostem and leaf decreased significantly as the Ca^2+^ concentration decreased. Furthermore, the P content in banana root, pseudostem and leaf and the S content in banana root increased significantly. It is possible that insoluble Ca_3_(PO_4_)_2_ and CaSO_4_ will be formed when Ca^2+^ coexists with PO_4_^3−^ or SO_4_^2−^. Therefore, the absorption efficiency of P and S in banana will significantly increase after suffering Ca^2+^ deficiency, resulting in the P and S accumulation increase in banana organs [19]. In addition, our study showed that the decrease of Ca^2+^ concentration greatly influenced the Mg and the K accumulations in banana.

### 3.3. Low Ca^2+^ and Ca^2+^ Deficiency Treatments Significantly Inhibited the Expression of Many Ca^2+^-ATPase Genes

Studies have shown that Ca^2+^-ATPase exists in all membrane systems of plant cells, and the expression of Ca^2+^-*ATPase* is tissue specific [37]. In our study, each member of the Ca^2+^-*ATPase* family was expressed in at least one organ. *MaECA4*, *6* and *MaACA7*, *12* were highly expressed in root, pseudostem, corm, and leaves, which was consistent with the expression of *AtACA8* and *AtACA10* [4], indicating that the function of these genes differed significantly. In our study, *MaACA4* and *MaACA11* were found to be specifically expressed in banana leaves, and *MaECA2* was specifically expressed in pseudocorm, indicating that their functions varied in different organs.

Ca^2+^-ATPases are considered to be required to maintain homeostatic levels of cytosolic Ca^2+^ and are widely believed to have roles in abiotic stresses via Ca^2+^ mediated signaling pathways. For instance, the expression of *AtACA8* was up-regulated when plants were under cold stress [38]. Similarly, the expression of *AtACA2* and *AtACA4* were enhanced under salt stress [6]. In the study, qRT-PCR results showed that under 2 mM Ca^2+^ condition, the expression levels of some banana Ca^2+^-ATPase genes, such as *MaECA4* and *MaACA7*, in banana root were both the highest, which was consistent with the previous study [3], while the expression of *MaECA4*, *MaECA6*, *MaACA1*, *MaACA7*, *MaACA8* and *MaACA10* declined under 1 mM and 0 mM Ca^2+^ concentration. The down-regulation of these banana Ca^2+^-*ATPase* genes indicated that they were responsive to low Ca^2+^ and Ca^2+^ deficiency conditions.

## 4. Materials and Methods

### 4.1. Identification of Banana Ca^2+^-ATPase Genes

The gDNA, CDS, and protein sequence files of *Musa acuminata* var. DH-Pahang were downloaded from the banana genome database (https://banana-genome-hub.southgreen.fr/blast, accessed on 10 January 2021). The HMMER software (version 3.0) (Sean Eddy, Cambridge, USA) [39] was applied to identify banana Ca^2+^-ATPase proteins using the Hidden Markov Model (HMM) file of Cation_ATPase_C (PF00690) (download from http://pfam.xfam.org/, accessed on 10 January 2021) with *e*-value ≤ 1 × 10^−5^. The conserved domain database (CDD, https://www.ncbi.nlm.nih.gov/cdd, accessed on 10 January 2021) was applied for the further confirmation of Ca^2+^-ATPases. The protein length, molecular weight, isoelectric point (pI) and instability index of banana Ca^2+^-ATPases were predicted using ExPASy ProtoParam (https://web.expasy.org/protparam/, accessed on 12 January 2021). To know whether their encoded proteins contain signal peptide and transmembrane structure or not, SignaIP 3.0 Server (http://www.cbs.dtu.dk/services/SignalP-3.0/, accessed on 12 January 2021) and TMHMM Server v.2.0 (http://www.cbs.dtu.dk/services/TMHMM/, accessed on 12 January 2021) was applied, respectively. For the subcellular localization analysis of banana Ca^2+^-ATPases, Wolf PSORT (https://wolfpsort.hgc.jp/, accessed on 20 January 2021) was used. For the self- and pairwise-alignment of Ca^2+^-ATPase proteins, the website for blast from NCBI (https://blast.ncbi.nlm.nih.gov/Blast.cgi, accessed on 20 January 2021) was used using *e*-value ≤ 1 × 10^−10^ as criterion. MCScanX (version 0.8) (Athens, Greece) was used for the gene collinear relationship analysis of the Ca^2+^-*ATPase* family members [40]. 

### 4.2. Phylogenetic Analysis

For the phylogenetic analysis of Ca^2+^-ATPases, the protein sequences of Arabidopsis and rice Ca^2+^-ATPases were downloaded from NCBI (http://www.ncbi.nlm.nih.gov/, accessed on 10 January 2021). MEGA 6.0 (Tokyo, Japan) [41] were used for the multiple sequence alignment and phylogenetic analysis, respectively. The phylogenetic tree was constructed using the Neighbor-Joining (NJ) method under parameters of Poisson model, Complete deletion and 1000 bootstrap replicates and was visualized using EvolView [42] (https://www.evolgenius.info/evolview/, accessed on 10 January 2021).

### 4.3. Gene Structure and Conserved Motifs Analysis

For gene structure analysis of banana Ca^2+^-*ATPase* genes, GSDS (http://gsds.cbi.pku.edu.cn/, accessed on 30 September 2022) was used. The conserved motif analysis of banana Ca^2+^-ATPases was performed using MEME (http://meme-suite.org/tools/meme, accessed on 30 September 2022) [43]. Furthermore, the gene structures of banana Ca^2+^-*ATPase* genes and conserved motifs of their encoded proteins were visualized using TBtools software 3.0 (Guangzhou, China) [44].

### 4.4. Promoter Analysis of the Banana Ca^2+^-ATPase Gene Promoters

The 2000 bp sequences upstream of the start codon of each banana Ca^2+^-*ATPase* genes were extracted from the banana genome database using TBtools software 3.0.(Guangzhou, China), PlantTFDB (http://planttfdb.cbi.pku.edu.cn/, accessed on 10 February 2021) and PlantCARE (http://bioinformatics.psb.ugent.be/webtools/plantcare/html/, accessed on 10 February 2021) was used to predict the transcription factor binding sites (TFBSs) and the *cis*-acting elements on promoters under default parameters, respectively. 

### 4.5. Plant Materials and Treatment

The plant materials we used in this study are tissue-cultured ‘Tianbaojiao’ banana (*Musa acuminata* cv. Tianbaojiao) seedlings. To facilitate the rooting and growth of tissue-cultured seedlings, ten days’ Hoagland solution (with Ca^2+^ concentration of 4 mM) hardening [45] and *Serendipita indica* inoculation [46] were performed. Banana seedlings were then transferred into clean river sand in flower pots (with diameter and height of 14 cm and 11 cm, respectively), and cultured at 25 °C growth chamber with 60–80% relative humidity and 160 mol m^−2^s^−1^ of illumination for 12 h per day. Seedlings were fertilized with Hoagland solution (with Ca^2+^ concentration of 4 mM) twice a week. Uniform and well-growing river sand cultured ‘Tianbaojiao’ banana seedlings at the five-leaf stage were divided into four groups which were watered with Hoagland solution containing 4 mM, 2 mM, 1 mM and 0 mM Ca^2+^ twice a week [47,48], respectively. The composition of the Hoagland solution containing different concentrations of Ca^2+^ are shown in Table S1.

### 4.6. Measurements of Growth-Related Parameters and Microelement Contents

To study the influences of different concentrations of Ca^2+^ treatments on the banana plant growth and microelement accumulations, the root number (referring to the number of all primary roots) and length (referring to the length of the longest taproot of the plant), fresh weight of aboveground part and root were determined at six months post treatment. For each parameter, at least three replications were made. Moreover, inductively coupled plasma atomic emission spectrometry (ICP-MS AES) was applied for the determination of the P, K, Ca, Mg and S contents in leaf, pseudostem and root of banana seedlings from the four groups. 

### 4.7. Gene Expression Analysis

The TPM values of Ca^2+^-*ATPase* genes were selected from root, corm, pseudostem, and leaf tissues of banana, and generated the plots by the R package pheatmap (version 1.0.12) (Raivo Kolde, Tartu, Estonia). To investigate the expression patterns of banana Ca^2+^-ATPase genes under the treatments of different concentrations of Ca^2+^, quantitative real time PCR (qRT-PCR) was used. An RNAprep Pure Plant Kit (TIANGEN, Beijing, China) was used for the isolation of total RNA from the roots of banana seedlings from the four groups. High quality RNA was used as a template for the cDNA synthesis using PrimeScript™ RT reagent Kit with gDNA Eraser (Perfect Real Time) (Takara, Beijing, China) according to the manual. The generated cDNA was diluted fivefold for subsequent experiments. The PCR reaction conditions used were 95 °C for 30 s; 95 °C for 5 s, and 59 °C for 20 s (40 cycles). Relative gene expression levels were determined using the 2^−^^∆∆Ct^ method by using *MaCAC* as an internal reference [49]. Statistical analysis and figure drawing was conducted using IBM SPSS Statistics 21 (International Business Machines Corporation, Armonk, USA) and GraphPad Prism 6.01 (Graphpad Software Inc, San Diego, USA) software, respectively. The primers used in this experiment are shown in Table S2.

## 5. Conclusions

In this study, we identified 18 Ca^2+^-*ATPase* genes from *M. acuminata*, including six *MaEACs* and 12 *MaACAs*. The expansion of this gene family was predicted to be caused mainly by segmental duplications. The gene structures of the banana Ca^2+^-*ATPase* genes from the same subfamily and motif distributions in their encoded proteins were much more similar, and all the banana Ca^2+^-ATPase proteins were predicted to be plasma membrane located. A gene expression analysis showed that the expression of banana Ca^2+^-*ATPase* genes in different organs varied greatly, and most members showed higher expression under 4 mM and 2 mM Ca^2+^ treatments, but significantly decreased expression under low Ca^2+^ (1 mM) and Ca^2+^ deficiency (0 mM) treatments. Therefore, it was concluded that the decreased expression of banana Ca^2+^-*ATPase* genes might be closely related to related to the reduced Ca accumulation and plant growth retardation caused by low Ca^2+^ and Ca^2+^ deficiency.

## Figures and Tables

**Table 1:** Physicochemical properties of the banana Ca^2+^-ATPase family proteins.

**Gene ID**	**Gene Name**	**Chromosome Location**	**Length (aa)**	**Molecular Weight/Da**	**pI**	**Instability** **Index**	**GRAVY**	**Signal Peptide**	**Transmembrane** **Structure** **Number**	**Protein** **Subcellular** **Localization** **Prediction**
P-type Ca^2+^-ATPaseIIA (ECA)										
Ma02_g17850	*MaECA1*	chr02:24603794…24609861(+)	1064	116,317.60	5.28	35.82	0.089	NO	7	Plasma membrane
Ma03_g18840	*MaECA2*	chr03:24338682…24347596(−)	1059	115,754.37	5.36	37.30	0.095	NO	9	Plasma membrane
Ma04_g23800	*MaECA3*	chr04:25894068…25901262(+)	1051	116,158.69	5.40	33.61	0.131	NO	8	Plasma membrane
Ma06_g16960	*MaECA4*	chr06:11494289…11502009(−)	1058	115,980.52	5.19	36.63	0.078	NO	7	Plasma membrane
Ma06_g26060	*MaECA5*	chr06:26671312…26740153(+)	1000	109,753.56	5.87	36.37	0.222	NO	8	Plasma membrane
Ma10_g13570	*MaECA6*	chr10:26320460…26327228(+)	1059	115,636.05	5.26	36.29	0.098	NO	9	Plasma membrane
P-type Ca^2+^-ATPaseIIB (ACA)										
Ma01_g18810	*MaACA1*	chr01:14159363…14175495(+)	1095	119,281.68	8.36	38.10	0.050	NO	8	Plasma membrane
Ma03_g13430	*MaACA2*	chr03:10547060…10561232(−)	1090	119,195.56	8.27	36.89	0.040	NO	11	Plasma membrane
Ma03_g31290	*MaACA3-1*	chr03:33555522…33560125(+)	942	102,943.61	5.98	35.91	0.221	NO	6	Plasma membrane
	*MaACA3-2*		1016	110,612.29	5.84	34.99	0.207	NO	6	Plasma membrane
	*MaACA3-3*		1012	110,180.84	5.84	34.60	0.216	NO	6	Plasma membrane
	*MaACA3-4*		1017	110,723.47	5.84	35.53	0.213	NO	6	Plasma membrane
	*MaACA3-5*		1034	112,541.57	5.87	35.13	0.220	NO	6	Plasma membrane
Ma04_g05840	*MaACA4*	chr04:4360063…4378280(+)	1082	118,244.80	8.47	40.33	0.066	NO	7	Plasma membrane
Ma04_g10940	*MaACA5*	chr04:7723372…7730223(+)	1020	111,212.01	5.88	35.51	0.200	NO	6	Plasma membrane
Ma04_g10640	*MaACA6*	chr04:7560089…7563361(+)	1017	111,767.31	5.70	31.68	0.121	NO	7	Plasma membrane
Ma04_g39050	*MaACA7*	chr04:36293787…36300960(+)	1020	111,518.14	5.91	32.30	0.158	NO	6	Plasma membrane
Ma05_g07830	*MaACA8*	chr05:5750563…5759010(+)	1034	114,138.10	6.74	31.15	0.169	NO	10	Plasma membrane
Ma06_g18390	*MaACA9-1*	chr06:12494351…12501092(−)	1019	111,002.40	5.26	32.57	0.201	NO	6	Plasma membrane
	*MaACA9-2*		1012	109,834.23	5.22	34.65	0.242	NO	6	Plasma membrane
Ma07_g27150	*MaACA10*	chr07:33645382…33649713(+)	1024	112,321.63	5.85	34.05	0.091	NO	8	Plasma membrane
Ma09_g16370	*MaACA11*	chr09:11747981…11758304(−)	1035	114,334.58	8.30	30.90	0.144	NO	10	Plasma membrane
Ma09_g21740	*MaACA12-1*	chr09:33595832…33627266(+)	1078	117,956.48	7.28	38.83	0.070	NO	9	Plasma membrane
	*MaACA12-2*		1103	120,673.88	8.87	35.90	0.072	NO	9	Plasma membrane

## Data Availability

The authors confirm that the data supporting the findings of this study are available within the article and its Supplementary Materials.

## Supplementary Materials

The following supporting information can be downloaded at: https://www.mdpi.com/article/10.3390/ijms231911914/s1.
